# Episodic memory and aging: Benefits of physical activity depend on the executive resources required for the task

**DOI:** 10.1371/journal.pone.0263919

**Published:** 2022-02-18

**Authors:** Ilona Moutoussamy, Laurence Taconnat, Kristell Pothier, Lucette Toussaint, Séverine Fay

**Affiliations:** 1 Département de Psychologie, Université de Tours et de Poitiers, Centre National de la Recherche Scientifique, Centre de Recherches sur la Cognition et l’Apprentissage (UMR, 7295), Tours, France; 2 Département de Psychologie, Université de Tours, Psychologie des Âges de la Vie et Adaptation (EA 2114), Tours, France; 3 Département des Sciences du sport, Université de Tours et de Poitiers, Centre National de la Recherche Scientifique, Centre de Recherches sur la Cognition et l’Apprentissage (UMR 7295), Poitiers, France; Technion Israel Institute of Technology, ISRAEL

## Abstract

Physical activity has beneficial effects on executive functions and episodic memory, two processes affected by aging. These benefits seem to depend on the type of memory task, but only a few studies have evaluated them despite their importance in understanding aging. This study aimed to confirm that the benefits of physical activity on episodic memory in older adults vary according to the executive resources required by the memory task, comparing free recall and cued recall. Thirty-seven young adults and 37 older adults performed two memory tasks and an updating task. The two groups had a similar level of physical activity over the preceding 12 months, assessed by a questionnaire. Both the memory and the updating tasks were performed better by the younger than the older adults. A similar cueing effect was observed in the two groups. Physical activity was positively correlated with updating and free recall, but not with cued-recall, and only in older adults. Regression analyses indicated that physical activity accounted for 24% of the variance in free recall in older adults. Updating did not predict free recall (ns) when physical activity was entered in the analysis. The present results show that the benefits of physical activity vary with age and episodic memory task. Only free-recall performance, which relies on updating, seems to depend on physical activity, suggesting that the executive resources required for the task play an important role in the effect of physical activity on memory performance. This should be investigated in greater depth in subsequent studies.

## Introduction

It is well established that aging is marked by episodic memory loss (i.e., encoding, storing and retrieval of personal experiences within their temporal and spatial contexts [1]), which is one of the most common subjective concerns of older adults [2]. With the aging of the population, it is important to gain a better understanding of the episodic memory decline that accompanies aging, as well as protective factors. Craik [3] postulated that the age-related decrease in processing resources prevents the implementation of efficient encoding and retrieval processes. This difficulty can be reduced by providing environmental support, such as specific instructions, material or cues, thereby improving the memory performance of older adults and even reducing the age effect [4–7]. In fact, a memory task that provides environmental support (e.g., cued recall) requires fewer executive resources than one without such support (e.g., free recall) [8, 9].

Executive functions are “a set of general-purpose control mechanisms, often linked to the prefrontal cortex of the brain, that regulate the dynamics of human cognition and action” [10], and it is widely accepted that they also decline with aging [11]. Several meta-analyses confirm that advancing age has an influence on the executive functions in Miyake et al.’s model [12]: updating, inhibition and flexibility [13–15]. The executive hypothesis of aging suggests that cognitive changes are the result of an age-related decline in executive functions [16] due to their involvement in cognitive processes, including memory, especially in older adults compared to younger adults [17, 18]. Some studies suggest that the memory strategy used is linked to executive functioning during aging (e.g., [19, 20]). For example, in the retrieval phase of a Remember-Know paradigm, participants have to say if they remember the word from the study phase and in what context (i.e., *Remember* responses considered as an episodic recollection), or if they remember the word but without any encoding details (i.e., *Know* responses, based on familiarity). Bugaiska and collaborators [21] found a correlation only between executive performance and *Remember* responses, involving a memory retrieval strategy related to the specific context. More specifically, updating, as measured by the N-Back task, was the executive measure that correlated most with *Remember* responses, in both younger and older adults, but particularly the latter [22]. Moreover, updating has been found to be the executive measure that best explains the variance observed in the age effect on memory and *Remember* responses, which could be due to better initiation of encoding and retrieval strategies such as memory search and checking the correctness of the answer [22]. *Know* responses, which are based on familiarity, do not appear to be related to executive functioning. Finally, prefrontal grey matter volume and activation have been linked to the use of self-initiated memory strategies by older adults [23, 24]. Thus, the well-established age-related differences in memory performance could be explained by differences in executive functioning (e.g., [17, 18, 25–27] but for a review see [8]). Older adults seem to have difficulty spontaneously implementing effective memory strategies, because they require more executive functions [28–30] to select, monitor and organize information in order to improve memory [31].

The memory performance of older adults varies widely, due to individual differences notably related to cognitive reserve [32], whereby external factors, such as educational level, leisure or physical activities, optimize cognitive performance despite brain changes. Researchers have been interested for some time in how the cognitive skills of older adults in particular benefit from physical activity, defined as “any bodily movement produced by skeletal muscle that results in energy expenditure” [33]. Although many studies have reported the benefits of physical activity on cognition, and especially on executive functions (e.g., [34]), they have yielded conflicting results. This can be explained by methodological differences; for example, interventional studies, which are widely used, compare performance before and after physical exercise [35, 36], while cross-sectional studies evaluate physical activity using actimetry [37, 38] or questionnaires [39–43] to compare performance with that of a control group. Physical activity has been shown to have a positive influence on executive functions such as updating, inhibition and flexibility [44–46], as confirmed by a recent meta-analysis of interventional studies [47]. Several meta-analyses of interventional studies tend to confirm that physical activity slows down executive and memory decline in older adults (e.g., [34, 48, 49]), but others have yielded more mixed results (e.g., [50, 51]). These discrepancies can be explained by study design, with variable durations and types of intervention (see [52] for a chapter devoted to studies by type of physical activity). However, the regularity of physical activity, reflecting lifestyle or habits, has been shown to have benefits on cognition (e.g., [43]). It is difficult to estimate the impact of regular physical activity through interventional or cross-sectional studies using actimetry, as interventional studies are carried out over a few weeks or months, while actimetry is used over a very limited period and could also lead to changes in the behaviour of the person wearing the device. A physical activity questionnaire could be an interesting alternative, providing a rapid way of estimating the level of physical activity over a long time-span.

Some studies have tried to explain the divergent findings about the benefits of physical activity during aging by task complexity. Chodzko-Zajko et al. [53] observed a difference between older exercisers with low and high fitness levels (based on their cardiovascular fitness level) in three memory tasks—an auditory free-recall task and two recognition tasks (frequency and location)—that differed in terms of environmental support and therefore in the cognitive resources required. Recognition performance was comparable between groups, but free-recall performance differed significantly, with better performance by the high-level fitness group. Woo and Sharps [54] compared the performance of younger and older adults with low and high levels of exercise (based on self-reported physical activity) on a free-recall task with 40 items (pictures or words of common objects). Results showed an interaction between exercise and item type (word vs picture) only in older adults. In other words, older adults with a low level of exercise remembered fewer words than pictures, but this difference was not observed in higher exercisers. According to Woo and Sharps [54], this difference could be explained by the level of support provided by the stimulus; pictures provide sufficient environmental support to enable older adults to remember them correctly, whatever their level of exercise, whereas words, which do not provide this support and need more cognitive resources, are recalled better by people with a higher level of exercise. In other words, exercise does not seem to have an overall effect on episodic memory, but only on the most resource-demanding tasks in which appropriate processes must be self-initiated. However, none of these studies investigated whether the effect of physical activity on more complex tasks could be mediated by the executive resources required.

The current study aimed to determine whether the benefits of physical activity on episodic memory during aging depend on the executive resources required by the task, especially updating, by comparing free-recall and cued-recall performance. We hypothesized first that both memory and updating tasks would be performed better by younger than older adults. Secondly, we expected that updating would be correlated more with free recall than with cued recall, indicating that more cognitive resources are needed for the former. Thirdly, we predicted that physical activity would have greater benefits for older than younger adults, and for free recall than cued recall because of the greater cognitive resources required to retrieve the item and its context without environmental support. Finally, we expected that physical activity would be the strongest predictor of free-recall performance in older adults, because of its benefits on updating, which is particularly involved in this task.

## Method

### Participants and background measures

We conducted *a priori* analyses using GPower 3.1 to test the differences between our groups (two age categories; inter-group factor) and our memory tasks (two memory tasks; intra-group factor), with a medium effect size (f = 0.25), an alpha of 0.05 and a repeated measures correlation of 0.50. The results indicated that we needed to include at least 54 participants to achieve a power of 0.95.

Seventy-four healthy French-speaking adults took part in this experiment. They were divided into two groups: 37 younger adults (20–40 years old) and 37 older adults (60–80 years old). All the participants were screened for their level of education (number of years of education), vocabulary (Mill-Hill, [55]), depression and anxiety (HADS, [56]), and the older adults completed the Mini Mental State Examination (MMSE, [57]). Eligible participants were selected according to the following criteria: (1) absence of symptoms of depression or anxiety (cut-off for exclusion > 11 on one of the two subscales of the HADS), (2) to be free of medication that could affect intellectual abilities, (3) a score greater than 27 on the MMSE to reduce the risk of including older participants with cognitive dysfunction. Background measures and comparisons between groups are reported in **Table 1**. Independent t-tests were used to compare characteristics of younger and older adults. The younger adults had a higher educational level and the older adults had a higher vocabulary level, these two variables were therefore included in the statistical analyses in order to control their potential effects on our measurements. The two groups did not differ in levels of anxiety and depression. Male to female ratio was compared using a Pearson’s Chi-squared test, indicating similarity between our two groups: 56% of younger adults and 54% of older adults were female [Pearson Chi^2^ = 0.05; p = .82]. This study was approved by the ethics committee of the University of Tours (France), and all participants signed a consent form.

**Table 1:** Means (and SD) of participants’ characteristics by age group.

	Younger adults (n = 37) Mean (SD)	Older adults (n = 37) Mean (SD)	*t*_72_
**Age (years)**	25.68 (5.10)	66.54 (5.12)	
**MMSE (score/30)**	/	28.30 (1.02)	
**Educational level (years)**	12.62 (2.19)	11.22 (3.56)	2.04, p = .044
**Vocabulary (score/34)**	20.46 (5.08)	23.95 (6.16)	- 2.65, p = .009
**Anxiety level (score/21)**	4.78 (3.14)	3.91 (2.85)	1.24, p = .219
**Depression level (score/21)**	5.05 (3.11)	3.97 (2.91)	1.54, p = .127

### Material

#### Episodic memory tasks

Participants performed two counterbalanced memory tasks, differing only at the retrieval stage (free recall vs cued recall). We created two lists of 20 common words, containing 4 to 9 letters and differing in the first 3 letters, and which can be completed by at least 5 words including the target word. Words in the two lists were similar in terms of length (6.15 ± 1.18 letters in the first list and 6.05 ± 1.19 letters in the second; *t*_38_ = -0.22, *p* = .79), frequency of use (9406 ± 12716 for the first list and 10161 ± 8933 for the second; *t*_38_ = 0.27, *p* = .83) [58]. A sample of 181 adults, aged 20 to 69 (mean age = 28.47 ± 12.13) were asked to indicate the concreteness of each word on a scale from 1 (very abstract) to 5 (very concrete). The concreteness is also equivalent between the two lists (3.48 ± 1.31 for the first list and 3.73 ± 1.23 for the second; *t*_38_ = 0.63, *p* = .53). The two lists were counterbalanced across memory tasks to avoid a list effect.

#### Updating measure

Participants performed the 2-Back task [59] to measure updating. Thirty letters were presented one at a time, and the participants had to decide whether the letter presented was the same as the one presented 2 letters before. The score was the number of correct responses (maximum 28).

#### Habitual physical activity index

To assess habitual physical activity, we used the Baecke questionnaire [60], which measures three domains of physical activity over the last 12 months, yielding three indices: IAT (index of physical activity at work, 8 questions), IAS (index of physical activity and sports, 2 questions), and IAL (index of leisure activity such as walking, 3 questions). The current study focused on older retired adults, so the IAT does not provide data discriminating between our older participants. Thus, like Bigard et al. [61], we used only two indices of habitual physical activity, IAS and IAL, Questions and calculation formulae to obtain these two indices are reported in **S1 Table**. The physical activity intensity codes for Q1 (**S1 Table**) were based on the estimated energy expenditure of the activity and classified into the categories defined by Durnin and Passmore [62]. The habitual physical activity index corresponds to the sum of IAS and IAL; the higher the index, the more the participant engages in daily physical activity.

Each index of habitual physical activity showed a great reliability on a three months test-rest (*r* = 0.81 for IAS and *r* = 0.74 for IAL). Finally, different authors correlated each index with finer measures. IAS was positively correlated with weekly energy expenditure estimated by another questionnaire [63], with physiological parameters such as VO_2_ max and with muscular endurance [61]. IAS was also negatively correlated to fat mass [61]. Concerning IAL, a positive correlation was found with VO_2_ max and a negative correlation with BMI (body mass index, [61]). Thus, this questionnaire has many times been correlated with objective measures representing a person’s physical condition, which makes it an interesting tool for measuring habitual physical activity.

### Procedure

Memory tasks were performed using the following procedure. Encoding and interference phases were similar in the two tasks. In the encoding phase, participants were asked to read each word aloud and try to memorise it. A fixation cross appeared for 1000 ms to prepare the participant for the word, then the word was presented for 2000 ms followed by a blank screen for 3000 ms. Words were presented randomly. Participants performed a short interference task at the end of this phase (counting backwards for 20 seconds) to prevent any recency effect. The procedure for the retrieval phase differed in the two tasks; in the free-recall task, participants were asked to say as many words as they could remember, and in the cued-recall task, the first three letters (word-stem) of each encoded word were displayed to guide retrieval. For this task, a fixation cross was displayed for 1000 ms, followed by the word-stem for 1000ms. A question mark was then displayed for 4000 ms and participants were asked to complete the word-stem with a word presented during the encoding phase. Each memory task can be completed in approximately five minutes. These two memory tasks were counterbalanced: in each age group, half of the participants started with free recall and the other half with cued recall. Five minutes of break and exchanges separated the two memory tasks to limit interference. The dependent variable was the percentage of correctly recalled words in each memory task (free recall and cued recall). In the 2-back task, thirty letters were presented aloud, one at a time, every 5 seconds. For each letter, participants had to answer "yes" if the letter presented was the same as the one presented two letters before or "no" otherwise. Five minutes are needed to complete this task.

The procedure was carried out in the following order: signing a consent form, MMSE (older adults only), then the cognitive tasks (memory and updating), and finally the physical activity questionnaire, Mill-Hill and HADS.

### Statistical analyses

Statistical analyses were conducted using Statistica 13.3 software. To analyse differences in recall depending on age and memory task, a 2 (age-group) x 2 (retrieval task) GLM (with educational and vocabulary level as covariates) was conducted on the percentage of correctly recalled words. Next, to analyse the effect of age on updating performance and on physical activity, one-way ANCOVAs were carried out. Educational and vocabulary level were added as covariates in both of these ANCOVAs to avoid bias related to the differences between our two groups. Effect sizes for significant effects and interactions were calculated using partial eta squared (η_p_^2^). Correlational analyses (controlling for age) of habitual physical activity and cognitive measures (cued recall, free recall and updating) were conducted separately in each age group to assess associations between these variables. Finally, hierarchical linear regression analyses were performed to establish the priority of predictors (physical activity and updating) of memory task performance. Hierarchical regression is sometimes criticized for adding its potential for overfitting when too many predictors and not enough observations are included in the analysis [64]. However, it seems that having between 10 and 15 observations per predictor would allow a good estimation. Below these 10 observations per predictor, the estimates may be biased [64, 65]). In our case, we had only two predictors and 37 participants, or more than 15 observations per predictor. Thus, adding physical activity in our regressions allows us to improve the explanation of older adult’s free-recall performance.

## Results

### Age effect on memory, updating tasks, and on physical activity index

Means and standard deviations for the two memory tasks, updating and habitual physical activity index for each age group are presented in **Table 2**. The 2 (age-group) x 2 (retrieval task) GLM (with educational and vocabulary level as covariate) confirmed the main effect of age group on memory performance, *F* (1,70) = 26.90, *p* < .001, ηp^2^ = 0.28, indicating that older participants remembered significantly fewer words than younger participants. The results also revealed a retrieval task effect on memory performance, *F* (1,70) = 4.90, *p* < .05, ηp^2^ = 0.06, participants recalling significantly more words in cued recall than in free recall, suggesting that the latter is more difficult. No interaction was found between age group and retrieval task, *F* (1,70) = 1.09, *p* = .30. In other words, the effect of age group was similar for cued recall and free recall, and cueing had similar benefits for younger and older participants.

**Table 2:** Means (and SD) for the two memory tasks, updating and habitual physical activity index in each age group (younger vs. older adults).

	Younger adults	Older adults
**Free recall (percentage of correctly recalled words)**	45.54 (12.79)	30.67 (11.91)
**Cued recall (percentage of correctly recalled words)**	70.54 (11.71)	60.00 (17.24)
**Updating**	24.97 (1.24)	23.32 (2.29)
**Habitual physical activity index (total index)**	6.08 (2.30)	6.60 (2.65)
**IAS (index of physical activity and sports)**	3.30 (2.17)	3.51 (2.46)
**IAL (index of leisure activity)**	2.78 (0.71)	3.10 (0.78)

A one-way ANCOVA was conducted on the 2-Back score to analyse the effect of age on updating. We found an age-group effect on updating performance, *F* (1,70) = 11.03, *p* < .01, ηp^2^ = 0.14, with higher scores for the younger than the older group.

Then, the ANCOVA on physical activity index revealed that habitual physical activity level (total index) was similar in the two age groups, *F* (1,70) = 0.81, *p* = .37, ηp^2^ = 0.01. More precisely, analyses did not show an age-effect on IAS, *F* (1,70) = 0.09, *p* = .76, ηp^2^ = 0.00, but showed a significative difference in IAL between our two groups, *F* (1,70) = 4.22, *p* < .05, ηp^2^ = 0.06.

### Correlational analyses

The main objective was to understand the role of our variables (updating, physical activity) in our principal outcome (memory performance). Therefore, correlational analyses (with age, education and vocabulary partialled out) of habitual physical activity and cognitive measures (cued recall, free recall and updating) were conducted separately in each age group. In both groups, updating performance (i.e., 2-Back score) was marginally or significantly positively correlated with the proportion of words recalled in the free-recall task (*r* = 0.32, *p* = .06 for younger adults and *r* = 0.41, *p* = .02 for older adults), indicating that the higher the 2-Back score, the greater the proportion of words recalled, whereas these correlations failed to reach significance in the cued-recall task (*r* = 0.23, *p* = .18 for younger adults and *r* = 0.14, *p* = .41 for older adults). Thus, only the free-recall task was linked to updating, which supports the idea that free recall depends more on cognitive resources than cued recall, particularly in older adults. Habitual physical activity index was positively correlated with the percentage of words recalled in the free-recall task (*r* = 0.53, *p* = .001) and with updating performance (*r* = 0.57, *p* < .001) only in older adults (in young adults, *r* = 0.06, *p* = .75 for physical activity and free-recall performance; *r* = 0.15, *p* = .40 for physical activity and updating performance). The latter results indicate that the higher the level of physical activity, the better the performance of older adults on free recall and updating. Finally, the habitual physical activity index was not significantly correlated with cued recall in either younger (*r* = 0.12, *p* = .49) or older adults (*r* = 0.23, *p* = .18).

### Regression analyses

For older adults, the previous analyses show that only performance on the free-recall task was correlated with physical activity and updating. Stepwise regression analysis was carried out to determine which of the three following factors (age, physical activity and updating) was the best predictor of free-recall performance in older adults. First, we calculated the percentage of variance explained by each factor when entered alone: age accounted for 3.21% of the variance observed in free recall (β = -.18; *t*_35_ = -1.08, *p* = .29), physical activity accounted for 24.15% of the variance (β = .49; *t*_35_ = 3.33, *p* = .002) and updating performance explained 13.63% (β = .37; *t*_35_ = 2.35, *p* = .02). When entered together, only physical activity proved to be a significant predictor of the variance related to free-recall performance. The variance explained by updating was reduced to a non-significant 1.23% (β = .13; *t*_34_ = 0.75, *p* = .46). Thus, the variance related to updating on free-recall performance was reduced by 90.97% after partialling out physical activity.

## Discussion

The aim of this study was to examine whether the benefits of physical activity on memory during aging depend on the amount of executive resources required by the task. First of all, the results confirm the well-established age-related memory decline (for a review, see [66]), younger participants recalling more words than older participants, in both free-recall and cued-recall tasks. They also revealed a cueing effect, cued recall leading to better performance than free recall. These results confirm that free recall is more difficult than cued recall. No significant interaction with age group was found, both groups performing better in cued recall than free recall. While some studies have found a greater benefit of cueing for older groups, consistent with the environmental support hypothesis (e.g., [6, 7]), our study found a similar benefit for the two age groups. It is possible that the first three letters of the target word used for cued recall did not provide sufficient support to further improve the performance of the older adults. Indeed, Angel et al. [67] found a significant age effect on cued recall when using a 3-letter but not a 4-letter word-stem, indicating that the amount of support given during retrieval modulates the effects of age on episodic memory, and therefore that certain cues do not lead to a greater improvement of the performance of older than younger adults. However, in the present study, we wished to ensure that a free-recall memory task was more difficult than a cued-recall task, which was confirmed.

Our main aim was to understand whether the involvement of physical activity in the performance of older adults differed according to the executive resources required by the memory task. To test this hypothesis, we needed to confirm that one of the two memory tasks required more executive resources than the other. As expected, only the free-recall task was positively correlated with the updating task, marginally in younger adults and significantly in older adults, indicating that the more executive resources, the better the free-recall performance. However, our results show that the correlation between updating and free recall was only marginal in young adults. These results are in accord with those of Bouazzaoui et al.’s [18], who found that executive index was not correlated with free-recall task or cued-recall task (respectively, r = —.03 and r = .01) in young adults unlike what was observed in older adult. However, in line with the environmental support hypothesis [3], the cues provided during retrieval seem to reduce the involvement of self-initiated strategies and therefore the involvement of updating in memory performance. More precisely, Clarys et al. [22] found that updating is correlated with *Remember* responses and not with *Know* responses, suggesting that updating could be implicated in contextualization in episodic memory. Another study highlighted a modification of the *Remember* responses (and not the *Know* responses) when updating processes were already involved in another task, which would confirm that the participants did not succeed in contextualizing the item [68]. The current study highlighted a greater involvement of updating in free recall than in cued recall, which is the task requiring to retrieve the item and its context. The results also revealed different patterns of correlations between age group and memory task, although the level of physical activity was similar in the two groups. For the younger adults, no significant link was found between physical activity and updating or memory tasks, could suggest that physical activity did not play a primary role in the quality of memory. Studies investigating this issue have yielded divergent results; some found benefits of physical activity on cognitive, and particularly executive functions, in younger adults (e.g., [69, 70] but see [71] for a review), while others found a benefit only in older adults (e.g., [37, 72] but see [73] for a review with limited evidence for the link between physical activity and cognition in younger adults), with weaker or even non-existent benefits in younger adults. In other words, executive performance and cerebral integrity are preserved in young adults, which makes it difficult to demonstrate the beneficial effects of physical activity, unlike in older adults. Thus, even if there are beneficial effects of physical activity on executive functions in younger adults, this may not be a discriminant factor in executive performance. However, in the older adults, the population of interest for this study, a positive correlation was found between physical activity and updating, indicating that older adults with a higher level of physical activity would also be those with the best updating performance (e.g., [46]). Performance on the free-recall task was also positively correlated with physical activity, indicating that older adults who engage more in physical activity perform better on a difficult memory task such as free recall. Thus, in accordance with Chodzko-Zajko et al. [52] and Woo and Sharps [53], we found that the benefits of physical activity in older adults were particularly noticeable in the free-recall task, which does not provide any environmental support and requires more updating.

In view of the results of the correlation analysis, we focused on the performance of older adults in the free-recall task only, since no significant correlation was found for cued recall. Both physical activity and updating explained free-recall performance (24.13% and 13.63% of the variance of recall respectively), but when analysed together, only physical activity remained significant, and updating was reduced to a non-significant 1.23% of the variance. This indicates that updating ability no longer influenced free-recall performance after controlling for the level of physical activity. We postulate that updating plays a decisive role in the link between physical activity and memory; physical activity could have benefits on updating, which in turn would lead to better memorization. Many studies have highlighted the beneficial effect of physical activity on executive functions (e.g., [34, 48, 49]), which are more involved in free recall due to the lack of environmental support and are well known for their role in supporting memory performance during aging [17, 18]. These results are consistent with the hypothetical explanatory model of Loprinzi [74], in which physical activity has an effect on various functions, in particular executive functions, which in turn influence memory performance. Another explanation to consider is the possible existence of a two-way link between regular physical activity and executive function. People with a higher level of executive function would be more apt to perform regular physical activities (and most importantly, to persevere in this healthy behavior), which in turn serves to improve executive functioning [75, 76]. Thus, in our results, regular sporting older adults would be those who already have an important executive function, which would have been further strengthened thereafter.

Overall, the present results would confirm the positive link between physical activity and cognitive performance of elderly adults (e.g., [77]). They also highlight the importance of updating in the beneficial effects of physical activity on memory performance. Accordingly, we can assume that the more older adults engage in physical activity, the better their updating performance and therefore their memory performance in the most resource-demanding tasks. No link was found between physical activity and memory in younger adults, in accordance with other studies (e.g., [37]). Executive functions are less important in the memory performance of younger adults [17, 18]. Thus, while physical activity improves the executive functions involved in self-initiated memory processes in older adults, it is not surprising that no link was found between physical activity and memory in younger adults. These results need to be confirmed by further studies including different executive measures. Indeed, while updating seems to be the main executive function involved in self-initiated retrieval in episodic memory [22, 66], having a more general view with an executive index or identifying the role of each executive function would help understand the benefits of physical activity. Thus, further studies are needed to identify not only the executive functions on which physical activity acts, but also the type of tasks where these beneficial effects are visible. In addition, other variables could influence executive and / or memory performance such as many other cognitive reserve factors (educational level, social environment, sleep, curiosity, etc.) or even IQ. Educational level corresponds to one of the most often factor used to represent and study cognitive reserve and is particularly important in healthy behavior changes and adherence [78]. Vocabulary level could also influence the cognitive performance due to its known role in IQ and memory, but also the link between physical activity and language [79]. However, the results found here showed that, even under the control of educational level and vocabulary, the declared physical activity is still the factor most linked to the free-recall performance of the older adults, which allows us to partially rule out these hypotheses. Finally, we acknowledge the limitations of the physical activity questionnaire (e.g., self-reported responses based on memory), but this measure seems to provide the best way to estimate the regularity of physical activity. It is important to validate the use of questionnaire measuring physical activity and to generalize this use. In fact, epidemiological studies, around public health on a very large population, do not allow us to easily use objective measurements (e.g., VO_2_ max or actimetry). The questionnaire used must be easy for everyone to understand, quick and easy to use–criteria fulfilled by Baecke questionnaire [60]. Despite this, it is possible that the actual intensity differs between the two groups and that an objective measurement could have perhaps revealed a difference, over a shorter period (i.e., a greater intensity of physical activity in younger adults than in older adults, generally practicing less intense sports), but it is important to note that the important results are still highlighted in the group of the elderly. Objective and subjective measures should not thus be put in opposition or in competition with each other but should rather be seen as complementary. In our case, the questionnaire we used was correlated to different objective measures, including cardiovascular health (VO_2_ max), confirming its validity [60].

In sum, aging is marked by episodic memory changes, which are greater when the memory task is more complex and requires more executive resources. Physical activity appears to be positively associated with updating in old age. It therefore seems logical to think that cognitive tasks requiring more executive resources are the most sensitive to physical activity. In fact, physical activity is a better predictor of free-recall performance than updating, and the latter could mediate the beneficial effects of physical activity on episodic memory. Thus, we conclude that the benefits of physical activity vary with age and type of episodic memory task. It seems that the executive resources required for the task play an important role in the effects of physical activity on memory performance and thus merit further investigation in subsequent studies.

## Supporting information

S1 Table Questions and calculation formulae from Baecke questionnaire [60]. (DOCX)
