# Supplementary material for: Episodic memory and aging: Benefits of physical activity depend on the executive resources required for the task
Source: PLoS One. 2022 Feb 18;17(2):e0263919. doi: 10.1371/journal.pone.0263919 (PMC8856534; doi:10.1371/journal.pone.0263919)
Supplement: S1 Table — (DOCX) [file pone.0263919.s001.docx]

**S1 Table**

| **IAS (Index of Physical Activity and Sports)**  **IAS = [ Σ (intensity x duration x proportion) + Q2 ] / 2** | |
| --- | --- |
| Q1. Do you do any sport or physical activity?   - Which sport or physical activity do you do most often? - How many hours a week?   (< 1; 1-2; 2-3; 3-4; > 4)   - How many months a year?   (< 1; 1-3; 4-6; 7-9; > 9) | Intensity: 0.76; 1.26; 1.76  Duration: 0.5; 1.5; 2.5; 3.5; 4.5  Proportion: 0.04; 0.17; 0.42; 0.67; 0.92 |
| If you do another sport or physical exercise,   - What is it? - How many hours a week?   (< 1; 1-2; 2-3; 3-4; > 4)   - How many months a year?   (< 1; 1-3; 4-6; 7-9; > 9) | Intensity: 0.76; 1.26; 1.76  Duration: 0.5; 1.5; 2.5; 3.5; 4.5  Proportion: 0.04; 0.17; 0.42; 0.67; 0.92 |
| Q2. In my leisure time, I do a sport or physical activity:  never/seldom/sometimes/often/very often | 1; 2; 3; 4; 5 |
| **IAL (Index of Leisure Activity)**  **IAL = (Q3 + Q4 + Q5) / 3** | |
| Q3. In my leisure time, I watch TV or play video games:  never/seldom/sometimes/often/very often | 5; 4; 3; 2; 1 |
| Q4. In my leisure time, I walk:  never/seldom/sometimes/often/very often | 1; 2; 3; 4;5 |
| Q5. For how many minutes a day do you walk or ride a bike going to and from work, school or the shops?  < 5 / 5-15 / 16-30 / 31-45 / > 4 | 1; 2; 3; 4; 5 |
